# Segmenting communities as public health strategy: a view from the social sciences and humanities

**DOI:** 10.12688/wellcomeopenres.15975.1

**Published:** 2020-05-26

**Authors:** Agomoni Ganguli-Mitra, Ingrid Young, Lukas Engelmann, Ian Harper, Donna McCormack, Rebecca Marsland, Lotte Buch Segal, Nayha Sethi, Ellen Stewart, Marlee Tichenor

**Affiliations:** 1 Centre for Biomedicine, Self & Society, University of Edinburgh, Edinburgh, UK; 2 School of Social and Political Sciences, University of Edinburgh, Edinburgh, UK; 3 School of Literature and Languages, University of Surrey, Guildford, UK

**Keywords:** social justice, COVID-19, public health response, ethics, inclusion, disability, ethnicity, equity

## Abstract

On the 5th of May 2020, a group of modellers, epidemiologists and biomedical scientists from the University of Edinburgh proposed a “segmenting and shielding” approach to easing the lockdown in the UK over the coming months. Their proposal, which has been submitted to the government and since been discussed in the media, offers what appears to be a pragmatic solution out of the current lockdown. The approach identifies segments of the population as at-risk groups and outlines ways in which these remain shielded, while ‘healthy’ segments would be allowed to return to some kind of normality, gradually, over several weeks. This proposal highlights how narrowly conceived scientific responses may result in unintended consequences and repeat harmful public health practices. As an interdisciplinary group of researchers from the humanities and social sciences at the University of Edinburgh, we respond to this proposal and highlight how ethics, history, medical sociology and anthropology - as well as disability studies and decolonial approaches - offer critical engagement with such responses, and call for more creative and inclusive responses to public health crises.

## Introduction

On the 5th of May, a group of modellers, epidemiologists and biomedical scientists from the University of Edinburgh proposed a ‘segmenting and shielding’ approach to easing the lockdown in the UK over the coming months (Van Bunnick *et al*, 2020) ^1^. Their proposal, which has been submitted to the government and since been discussed in the media, offers what appears to be a pragmatic solution out of the current lockdown. The approach identifies segments of the population as at-risk groups and outlines ways in which these remain shielded, while ‘healthy’ segments would be allowed to return to some kind of normality, gradually, over several weeks. However, the ‘Edinburgh Proposal,’ as the Guardian ^2^ describes it, highlights how narrowly conceived scientific responses may result in unintended consequences and repeat harmful public health practices. Indeed, as the authors note, they are 'unaware of segmenting and shielding' being proposed as a major public health initiative previously. Although not using this precise terminology, the practices and policies suggested have a long history in colonial and 20th century responses to pandemics. As their University of Edinburgh colleagues, we argue that a better appreciation of, and engagement with the evidence provided by the humanities and social sciences, as well as greater disciplinary diversity, would better anticipate and hopefully avoid potential harm ^3^.

The proposed model discusses the ‘public health burden’ of COVID-19 without specific attention to public health ethics, and without acknowledgement of the long history of the detrimental effects of segmenting vulnerable populations. Public health ethics evaluate measures in terms of both their – stated and implicit – justifications for restricting individual freedoms for the collective good, as well as the paternalistic practices that restrict the individual freedoms of those who are being protected. It is not clear that either of these considerations has been systematically analysed and justified in a transparent manner. The model does not clearly evaluate whose interests and rights are being traded off. Nor does it consider the ethical implications of social determinants of health, or of existing health inequalities. Insights from medical sociology and anthropology have shown us that social solutions to public health harms need to address how social inequalities will be exacerbated in the context of public health emergencies. These disciplines also illustrate how the sustainability of ‘behavioural’ and cultural practices in the context of health interventions are dependent on investment from, and engagement with, affected communities. Moreover, the model does not reflect on the well-documented history of public health motivated segmentation of societies with its deep roots in colonialism. One could consider the history of cordon sanitaires drawn around white colonial hill stations in India and African countries, designed to protect white settlers from malaria ^4^. Or we could look to the histories of social segregation in the nineteenth century history of cholera in the UK, of quarantining San Francisco’s Chinatown during the third plague pandemic in the US and, perhaps most obviously, of risk grouping along the lines of sexual identity in HIV ^5^. Questions of segmenting populations along measures of perceived vulnerability have always informed public health practices. However, and more importantly, the very same histories have also shown how arbitrary, contingent and problematic concepts of vulnerability can be. The history of health ethics is rife with examples of vulnerability being used to implement unjustified protectionist and restrictive measures, as well as problematically labelling and stereotyping entire groups ^6^, thus further silencing them.

The proposed model frequently draws on the notion of vulnerability. We suggest that if used as a cornerstone of public health measures, it needs far more careful and nuanced deployment. ‘Vulnerability’, as normative justification for such a policy, ignores the multifaceted lives that are lived behind this concept. Vulnerability does not merely cover susceptibility to ill-health or disease. It also means being left unable to protect oneself and others against harm, as a result of social and structural inequalities, historical and current oppression and marginalisation.

## Vulnerability and segments of blame

Van Bunnik *et al.* base their mathematical model on assumptions about ‘vulnerable’, ‘shielders’ and ‘non-vulnerable’ members of the population. Without asking them, it’s not possible to know what kind of society they imagined when they made these assumptions, but we can consider what kind of society the model might bring to mind and wonder about the implications of this for policy.

Crucially there is the vulnerable-shielder dyad, which they assume exists in a 1:1 ratio ^7^. For mathematical models this might be abstract and population-based, but this will have to be translated into reality. Is it households of elderly couples or couples of working-age but both living with chronic illness? In which case are the vulnerable not *also* shielders in this dyad? Are they bearing in mind multiple generation households, with one or two vulnerable members, and three or four shielders? Who are all of these shielders in this context? Paid live-in carers? If a vulnerable person lives alone, do they embody a singular vulnerable self-shielding person? Or do they have a carer who visits and then who is the visiting carer, who does that carer live and work with, what social group do these carers predominantly come from, and are all of their household members and colleagues also shielders? What of care homes? Will these now have as many care workers in them all the time as they do residents who need shielding? All of these questions suggest that we cannot straightforwardly “segment” real human beings into these different categories.

Another concern in the model is a shift from a society that takes responsibility for the vulnerable to one in which the responsibility is now held by these individualised shielders. The language of protection, complete with its military metaphor, signals the weight of this responsibility. What will happen when the shield inevitably breaks from time to time? Will the shielders be blamed or blame themselves if the vulnerable person dies from COVID-19? Accounts of the history of AIDS or cholera have shown in painstaking detail how the attribution of vulnerability incorporates geographies of blame ^8^, how segmentation tends to track existing socio-economics divisions and how it exacerbates persisting injustices ^9^. 

This shift from society to the individual also makes invisible that there is already a shield in place which has not been adequately acknowledged. Lockdown, as a public health measure, is one which protects some members of society and not others. Those who can afford to stay at home do so, whereas others – “key workers” – form the shield around those in lockdown by providing essential services. Without systematic consideration of context, using a simple metric such as age and selected underlying health conditions as justificatory cornerstone of severely restrictive public health measures is problematic ^10^. Ageism has already been picked up by various commentators ^11^ in relation to the proposed model. In what follows we highlight further important social and ethical considerations.

## Ableism and chronic conditions

As currently framed, orienting public health responses around ‘the healthy’ erases decades of work in disability activism and legislative efforts, which identified how exclusion is built into the structural and social conditions of our environment. Ableism is the socio-political act of normalising exclusion from society, as if segregation is a collective form of existence to which we willingly agree. Like ableds, those of us with disabilities and/or chronic illnesses agree to isolate ourselves away as a form of protection from COVID-19. Of particular concern, however, is that the proposal does not lay out at what point those of us considered at high risk of being seriously ill should we contract the virus will be able to return to the outside world. Do we structure our collective social and political contract on the grounds of segregation of some for public life to continue and for the NHS to survive? ^12^ Do we want to arrive at a division between “sanitary citizens” and “unsanitary subjects”? By focusing on high risk populations, we avoid issues of chronic underfunding, of austerity-based policies and of ongoing inequalities as the virus spreads disproportionately among certain populations.

We raise these questions about the proposed model in the hope of drawing urgent attention to the increasing ease with which this imagined population may be left at home for an indeterminate amount of time. And, here, we would draw attention to the fact that this category of people - at high risk of being seriously ill should COVID-19 be contracted - is large, diverse and differently abled. Moreover, this category of people includes those who work, care and take part in everyday life. Will an easing of lockdown while segregating these people mean those segregated will be subject to different rights, including protection from employers’ demands to return to work? How will these segmenting and shielding practices serve as obstacles to the inclusive and compassionate society we want to create?

## Health and social inequalities

Although current public health measures have overwhelmingly identified those with particular health conditions as at risk, social and structural inequalities exacerbate and heighten the effects of illness at times of crisis ^13^. This is particularly true of Black, Asian and minority ethnic communities in the UK in the context of COVID-19, and has been well documented ^14^. There is considerable evidence that the UK was unprepared in terms of anticipating the severe consequences of the pandemic on BAME and other minority communities ^15^. In the proposal to implement a segment-and-shield approach to relaxing a lockdown, we stand to repeat the same mistakes. Additionally, given that BAME workers are considerably over-represented in our current ‘shield’, as NHS staff, carers and key workers, how should we reconcile the potential risks and harms associated with being both more vulnerable in terms of risks, and having to nevertheless, carry out essential work ^16^ that keeps the economy and life itself going?

While the proposal uses the broad term of vulnerable in relation to COVID-19, it does not discuss the impact of this virus on populations who may not be able to self-isolate, who might not have control over accommodation, or who may be at the whim of a local or national authority. Consider the effects on individuals and communities who have left contexts of oppression and violence, conflict, forced displacement and forced confinement. These are also potentially vulnerable individuals whose mental and physical well-being might be worsened and sacrificed in the process ^17^. Implementation of public health policies, which fail to take into consideration these health needs, have already had deadly consequences ^18^.

Global health emergencies, as well as public health responses have demonstrably gendered effects ^19^. As documented by Gelder *et al.* last month ^20^, domestic violence soars as a consequence of lockdown measures. Those who have been adversely affected by the lockdown, including LGBT individuals living in hostile households ^21^, are not accounted for in the consideration of further segmentation and confinement. Despite the many testimonies related to the gendered effects of the care burden in lockdown ^22^, very little attention is being given to this in the proposed models going forward. Individual families might be left with difficult decisions related to how and when to send children to school, who will go back to work, especially if they also have to take additional precautions to protect elderly or chronically ill family members who might be living in the same household. The additional care and emotional burden will almost certainly also be gendered.

Considering how to trade off the worth of a biological life against trying to avoid a lifetime of complex trauma for those children and kin belonging to the perceived vulnerable might be an unconscionable task, but ignoring these questions entirely will most certainly result in further unanticipated harms. The effects of the existing measures have been considerable on mental and physical wellbeing. It is likely that we will be seeing the repercussions ^23^ of both the pandemic, as well as our public health responses in the patterns arising in social determinants of health for decades. Implementing measures without taking into account the lessons learnt from history and without the invaluable knowledge gathered in the biomedical humanities, will most certainly exacerbate these patterns.

## Creative and inclusive responses to public health crises

The history of infectious disease and global health emergencies have shown that while some advocate for responses which restrict rights and movement, others have responded with creativity to embrace harm reduction approaches that centre care for the other. Well documented responses to HIV - in the UK and globally - show how creative responses in sexual and social practices challenged (and proved more sustainable) in the face of abstinence and quarantine messages; needle exchange, condom use, creative sexual practices and community-based approaches to sharing messages no doubt reduced HIV transmission in the years before treatment became available ^24^. Other epidemics, such as Ebola, have shown how ignoring the knowledge and concerns of communities affected can have disastrous consequences and only exacerbate health and social crises ^25^. Any response needs to increase its engagement with affected communities to explore what works together as a means of overcoming this current challenge ^26^. People are willing to make sacrifices for the health of others and for their own health; this collective response, however, needs to care for all and take into consideration the multiple physical, social and mental health needs of all. While we recognize the importance of understanding what difficult decisions need to take place, those of us directly affected by these measures need to be involved, as advocated by the Greater Involvement of People Living with HIV (GIPA) Principle: nothing about us without us ^27^.

As we begin to consider easing lockdown, we need to embed consideration and care for all in any response. Who belongs to the healthy population that can be a part of lockdown restrictions? We cannot start from a universal position of young, healthy, white and able bodies where the vulnerable can be segmented and segregated, but must start from a position that assumes vulnerability in all and build a system that accounts for this. Any response - modelled and implemented - needs to be grounded in the ethics and practice of social justice. This includes access to health resources (including ongoing access for those illnesses unrelated to COVID-19), decent and affordable housing, universal income and safety from harm. Our primary concern is that this proposal demonstrates the risks of moving rapidly from scientific evidence to policy proposals, without full consideration of the social and ethical dimensions of decisions, or engagement with communities expected to comply with and affected by such policies. Such engagement must happen now, during the crisis, especially given the uncertainty of the virus and its ongoing impact on us all.

## Data availability

No data is associated with this article.

## Notes

^1^ Van Bunnik *et al* (2020) Segmentation and shielding of the most vulnerable members of the population as elements of an exit strategy from COVID-19 lockdown https://www.wiki.ed.ac.uk/display/Epigroup/COVID-19+project?preview=/442891806/447360858/van%20Bunnik%20et%20al.%20SS%20manuscript%20050520.pdf [accessed 11 May 2020]

^2^ Sample & Mason (2020) UK could relax lockdown for millions if over-70s are shielded, scientists say, The Guardian 5 May https://www.theguardian.com/society/2020/may/05/longer-lockdown-for-over-70s-would-allow-fewer-restrictions-for-rest-of-uk-scientists-suggest

^3^ While we note that the Scottish Government’s ‘Coronavirus (COVID-19): Framework for decision making’; acknowledges some of these harmful assumptions, it falls short of directly addressing how it will address these inequalities which place increased restrictions (and burden) on those who are shielding.

^4^ Sacareau, I (2007) Himalayan Hill Stations from the British Raj to Indian Tourism. European Bulletin of Himalayan Research. 31 Spring 2007: 30-45.

^5^ Shah, Nayan. *Contagious Divides: Epidemics and Race in San Francisco’s Chinatown*. Berkeley: University of California Press, 2001.

^6^ Levine *et al*, ‘The Limitations of “Vulnerability” as a Protection for Human Research Participants’, Am J Bioeth. 2004 Summer;4(3):44-9.

^7^ Van Bunnik *et al*, 2020

^8^ Farmer (1992) AIDS and Accusation: Haiti and the Geography of Blame, University of California Press

^9^ Briggs & Mantini Briggs (2004) Stories in the Time of Cholera, on the cholera outbreak in Venezuela, University of California Press

^10^ See list at the end of the proposal ( https://www.wiki.ed.ac.uk/download/attachments/442891806/van%20Bunnik%20et%20al.%20SS%20manuscript%20050520.pdf?version=1&modificationDate=1588665237000&api=v2) and NHS Digital ( https://digital.nhs.uk/)

^11^ J. Farrar https://www.pressreader.com/uk/the-scotsman/20200505/281767041389881

^12^ Briggs & Mantini Briggs 2004

^13^ Higher death rate in poorer areas, ONS figures suggest (2020) BBC news https://www.bbc.co.uk/news/uk-52506979 [accessed 11 May]

^14^ Siddique (2020) British BAME Covid deaths more than twice that of whites, The Guardian 1 May https://www.theguardian.com/world/2020/may/01/british-bame-covid-19-death-rate-more-than-twice-that-of-whites [accessed 11 May]

^15^ Qureshi *et al* (2020) Submission of evidence on the disproportionate impact of COVID-19, and the UK government response, on ethnic minorities in the UK

https://ghpu.sps.ed.ac.uk/wp-content/uploads/2020/04/Qureshi-Kasstan-Meer-Hill_working-paper_COVID19-ethnic-minorities_240420.pdf

^16^ Molloy (2020) Don’t buy the lockdown lie - this is government business as usual, Open Democracy, https://www.opendemocracy.net/en/oureconomy/dont-buy-the-lockdown-lie-this-is-a-government-of-business-as-usual/

^17^ Ahmad (2020) ‘Conflict and COVID-19- A War Storyteller’s Narrative of Violence and Silence during a Pandemic’: https://www.ghe.law.ed.ac.uk/conflict-and-covid-19-a-war-storytellers-narrative-of-violence-and-silence-during-a-pandemic/

^18^ Paterson (2020) Fury after Syrian asylum seeker found dead in Scottish Hotel, Glasgow Times 10 May https://www.glasgowtimes.co.uk/news/18439256.amp/?__twitter_impression=true&fbclid=IwAR2H0QEt7PYVhNR1pT7zDDDQi6Qz4zn-C7Xq8BZ8my1qoCZT-rdga21svns

^19^ Wenham *et al* (2020) COVID-19: the gendered impacts of the outbreak, The Lancet 395:10227, p846-848 https://www.thelancet.com/journals/lancet/article/PIIS0140-6736(20)30526-2/fulltext

^20^ Gelder *et al*.(2020) COVID-19: Reducing risk of infection might increase the risk of intimate partner Violence, The Lancet 21:100348. https://www.thelancet.com/journals/eclinm/article/PIIS2589-5370(20)30092-4/fulltext

^21^ Kelleher (2020) LGBT+ helpline sees calls double as queer people fear being left with abusive families during coronavirus lockdown, Pink News 27 March https://www.pinknews.co.uk/2020/03/27/lgbt-foundation-helpline-increased-calls-coronavirus-covid-19-pandemic-queer-gay-mental-health/

^22^ Ferguson (2020) ‘I feel like a 1950s housewife’: how lockdown has exposed the gender divide, The Guardian 3 May https://www.theguardian.com/world/2020/may/03/i-feel-like-a-1950s-housewife-how-lockdown-has-exposed-the-gender-divide [accessed 11 May]

^23^ Holmes *et al* (2020) Multidisciplinary research priorities for the COVID-19 pandemic: a call for action for mental health services, Lancet Psychiatry https://www.thelancet.com/journals/lanpsy/article/PIIS2215-0366(20)30168-1/fulltext

^24^ Kippax & Race (2004) Sustaining Safe Practice: 20 years on, Social Science and Medicine, 51: 1, 1 - 12.

^25^ Ebola Response Anthropology Platform http://www.ebola-anthropology.net

^26^ Marsten, Renedo & Miles (2020) Community participation is crucial in a pandemic, The Lancet, https://doi.org/10.1016/S0140-6736(20)31054-0

^27^ UNAIDS (2007) The Greater Involvement of People Living with AIDS http://data.unaids.org/pub/briefingnote/2007/jc1299_policy_brief_gipa.pdf

