# Supplementary material for: Segmenting communities as public health strategy: a view from the social sciences and humanities
Source: Wellcome Open Res. 2020 May 26;5:104. [Version 1] doi: 10.12688/wellcomeopenres.15975.1 (PMC7309410; doi:10.12688/wellcomeopenres.15975.1)
Supplement: Peer review history

# Peer review: Reviewer response for version 1

**DOI:** 10.21956/wellcomeopenres.17520.r38844

**Published:** 2020-06-19

**Authors:** Eric Mykhalovskiy

**Affiliations:** 1 Department of Sociology, York University, Toronto, ON, Canada

Ganguli-Mitra and colleagues have written an open letter that offers an important critique of the ‘segmenting and shielding’ approach proposed by epidemiologists, modellers and biomedical scientists from the University of Edinburgh (Van Bunnick *et al.*, 2020)[1] as a way to transition out of UK “lockdown” measures introduced in response to COVID-19. The paper draws on arguments from the social sciences, humanities, population and public health ethics, history, critical disability studies, and other sources of knowledge to raise serious concerns about the model’s potentially harmful consequences. Ganguli-Mitra and colleagues invite readers to consider the limitations of the approach given its overcommitment to abstraction, inattention to the social determinants of health and health inequities, and the failure of the modellers to take into account structural inequalities based on intersections of race, class, gender, disability, and sexual orientation that not only heighten people’s vulnerability to COVID-19 but that place them at greater risk of harm by the very public health measures meant to respond to the pandemic.

 Overall I find the letter convincing. I have some suggestions that might clarify, extend and deepen the argument.

 1) I suspect readers would benefit from a pithy statement about the nature of the model proposed. The open letter’s argument would be strengthened and readers would be more able to fully engage with it were the authors to provide a short “lay” account of the model itself—what kind of knowledge is it? What forms of expertise does it draw on? What forms of calculation does it rely on and how does it propose to work? This need not be long or overly complex.

 2) Given the many significant and convincing concerns posed by Ganguli-Mitra and colleagues about the proposed segmenting and shielding approach, one wonders why it and similar modelling exercises have captured the attention of policy makers from multiple national jurisdictions in the wake of the COVID-19 pandemic.  Ganguli-Mitra might offer some thoughts on why such models are so popular right now. This might be done in such a way that creates a stronger connection between the open letter and emerging social science writing about modelling and the public health and policy response to the COVID-19 pandemic.[2] How does their critical analysis of the segmenting and shielding approach also contribute to broader social science critiques of the centrality of modelling within the response to the COVID-19 pandemic?

 3) The authors turn to historical work on the colonial legacy of social medicine to discuss “how arbitrary, contingent and problematic concepts of vulnerability [upon which the model relies but does not specify] can be” and to suggest connections between the segmenting and shielding approach and the history of cordon sanitaires used to “protect white settlers from malaria” (Ganguli-Mitra 2020, p. 3 of 6). Given their critique of how the science of pandemic response can result in interventions that harm people living in circumstances of social inequality and marginalization, one might add a further lesson from the history of epidemics in the UK that suggests important connections between current and past public health practice, namely, that the very first efforts on the part of the state to curtail individual liberties in the name of protecting the public’s health targeted “the bodies of those who were least able to protest” (Porter and Porter 1988, p. 107).[3]

 4) Finally, Ganguli-Mitra have stayed relatively close to the established lineaments of what might be termed social science’s predilection for “negative critique”(Mykhalovskiy *et al*. 2019)[4]—pointing out what is failing, what is wrong, and so on. At the same time, they do suggest alternative ways of addressing the pandemic that is based on “engagement with affected communities to explore what works together as a means of overcoming this current challenge” (p. 5 of 6). One wonders if this might be pushed further. Are there examples of approaches from the UK or other relevant jurisdictions that are based in community engagement that might be described? If one accepts that the current challenge is one of moving past existing public health restrictions that have created lockdown, what, more specifically, might a process of easing lockdown that is informed by social science, history, humanities and so on, look like?       

 [1] Van Bunnik *et al* (2020) Segmentation and shielding of the most vulnerable members of the population as elements of an exit strategy from COVID-19 lockdown https://www.wiki.ed.ac.uk/display/Epigroup/COVID-19+project?preview=/442891806/447360858/van%20Bunnik%20et%20al.%20SS%20manuscript%20050520.pdf [accessed 19 June 2020]

 [2] Rhodes, T., & Lancaster, K. (2020). Mathematical models as public troubles in COVID-19 infection control: following the numbers. *Health Sociology Review*, 1-18; Rhodes, T., Lancaster, K., & Rosengarten, M. (2020). A model society: maths, models and expertise in viral outbreaks; Montgomery, Catherine & Engelmann, Lukas. (2020). *Epidemiological publics? On the domestication of modelling in the era of COVID-19*. Retrieved June 19, 2020, from Somatosphere Web site: http://somatosphere.net/2020/epidemiological-publics-on-the-domestication-of-modelling-in-the-era-of-covid-19.html/; Hinchliffe, Steve. (2020). *Model evidence – the COVID-19 case*. Retrieved June 19, 2020, from Somatosphere Web site: http://somatosphere.net/forumpost/model-evidence-covid-19

 [3] Porter, D. and R. Porter (1988). “The enforcement of health: The British debate.” In E. Fee and D.M. Fox (Eds.) *AIDS: The Burdens of History.* Berkeley: University of California Press pp. 97-120.

 [4] Mykhalovskiy, E., Frohlich, K., Poland, B. Ruggiero, E., Rock, M., and Comer, L. (2019). Critical social science with public health: Agonism, critique and engagement. *Critical Public Health*, 29(5): 522-533.

Does the article adequately reference differing views and opinions?

Yes

Are all factual statements correct, and are statements and arguments made adequately supported by citations?

Yes

Is the Open Letter written in accessible language?

Yes

Where applicable, are recommendations and next steps explained clearly for others to follow?

Yes

Is the rationale for the Open Letter provided in sufficient detail?

Yes

Reviewer Expertise:

sociology of health and illness, sociology of public health

I confirm that I have read this submission and believe that I have an appropriate level of expertise to confirm that it is of an acceptable scientific standard.

# Peer review: Reviewer response for version 1

**DOI:** 10.21956/wellcomeopenres.17520.r38843

**Published:** 2020-06-16

**Authors:** Ayo Walberg

**Affiliations:** 1 Department of Anthropology, University of Copenhagen, Copenhagen, Denmark

This is an important rejoinder to a proposal put forth by a group of epidemiologists which suggests segmented shielding of vulnerable groups as a way out of lockdown. The authors argue that such proposals must be submitted to scrutiny from a public health ethics perspective. As it stands, the authors argue that pursuing a segmented shielding approach would in fact intensify stigmatisations while also disregarding the difficulties that huge numbers of people have in isolating themselves. I have a couple of recommendations for the authors.

 When discussing the ableism that is inherent in suggestions that persons living with chronic conditions must be responsible for isolating and shielding themselves, the authors need to point out that whatever approach that might be pursued, living with a chronic condition just got that much more complicated due to COVID-19. So it is not just ableism, it is also that shielding persons living with chronic conditions ironically will put them at increased risk of their known conditions.

 Also I would urge the authors to be more concrete in terms of outlining ways forward for those who have been identified most at risk of COVID-19. There is research on living with risk that can be helpful here. So, if not segmented shielding, how would an approach other than total lockdown based on public health ethics be feasible?

Does the article adequately reference differing views and opinions?

Yes

Are all factual statements correct, and are statements and arguments made adequately supported by citations?

Yes

Is the Open Letter written in accessible language?

Yes

Where applicable, are recommendations and next steps explained clearly for others to follow?

Partly

Is the rationale for the Open Letter provided in sufficient detail?

Yes

Reviewer Expertise:

chronic living, social studies of medicine, anthropology

I confirm that I have read this submission and believe that I have an appropriate level of expertise to confirm that it is of an acceptable scientific standard.
